# How Much Is Winning a Matter of Luck? A Comparison of 3 × 3 and 5v5 Basketball

**DOI:** 10.3390/ijerph20042911

**Published:** 2023-02-07

**Authors:** Gergely Csurilla, Zoltán Boros, Diána Ivett Fűrész, András Gyimesi, Markus Raab, Tamás Sterbenz

**Affiliations:** 1 Institute of Economics, Centre for Economic and Regional Studies, 1097 Budapest, Hungary; 2 Sport Economics and Decision Making Research Centre, Hungarian University of Sports Science, 1123 Budapest, Hungary; 3 Faculty of Business and Economics, University of Pécs, 7624 Pécs, Hungary; 4 Institute of Psychology, German Sport University Cologne, 50933 Cologne, Germany; 5 School of Applied Sciences, London South Bank University, London SE1 0AA, UK

**Keywords:** International Basketball Federation (FIBA), World Cup, uncertainty of outcome, competitive balance, chance

## Abstract

Background: The comparison of team sports based on luck has a long tradition and remains unsolved. A contrast between the new Olympic format three-on-three (3 × 3) and five-on-five (5v5) forms of basketball has never been analyzed and provides a comparison within the same form of sports. Methods: We developed a new method to calculate performance indicators for each team and invented the Relative Score Difference Index, a new competitive balance indicator that allows the comparison of luck in the two basketball forms for both men and women. We collected game-level data about 3 × 3 and 5v5 from the World Cups held between 2010 and 2019 (*N* = 666). Luck was defined as the difference between the expected and the actual outcomes of games. Using the basketball World Cup data, we applied the Surprise Index, ran probit regression models, and compared the basketball forms on the goodness-of-fit of the models. Results: As we predicted, there are differential effects of luck between game formats and sex, such that the 3 × 3 form depends more on luck and women’s games are less influenced by luck when compared to men’s games. Conclusion: Coaches may better understand the differences between the two forms and sexes regarding luck if they are aware that the 3 × 3 and men’s competitions are usually more influenced by luck. The findings provide a leverage point for testing new performances and competition balance indicators and will acknowledge the number of games we enjoy watching.

## 1. Introduction

The basketball form of three-on-three (3 × 3) came to the forefront of interest in 2017 when it was added to the Olympic program. Its Olympic debut would have been at the Tokyo 2020 Games, which have been postponed because of the COVID-19 pandemic. In 2021, the first Olympic 3 × 3 event was finally held, where Latvia won the men’s event and the United States the won event for women.

The 3 × 3 form of basketball differs markedly from the five-on-five (5v5) version in many respects [1]. The most important difference is probably the size of the court; the 3 × 3 game is played on a half-court with one basket, whereas the 5v5 utilizes the full court and has two baskets. The available time for attack is also shorter: 12 s in 3 × 3 versus 24 s in 5v5. Because of the narrower/smaller area and the limited time for the offense, there is less player movement in 3 × 3 overall, but the game is more intense. The additional two players in 5v5 make the play more difficult and unpredictable, with the demands of coordinating the movement of five players requiring complex tactics. Additionally, one of the most remarkable rules in 3 × 3, which leads to a difference in intensity, is the lack of stopping the game after scoring. After scoring in 5v5, a player must leave the court and then bring the ball back into play; in 3 × 3, the match continues without a break and with a defensive rebound. The lack of a break leads to continuous changes in the defensive and attacking roles, which requires immense concentration from the players.

The comparison of sports or element of sports based on the level of luck or probability of winning is not novel, and several studies have investigated this topic [2,3,4,5,6,7,8]. There are lucid reasons behind the popularity of luck studies in sport [2]. Sports contests are relatively isolated systems, and the games are replicated over time and under the same rules. Consequently, a comparatively large amount of data is generated, which enables researchers to analyze the statistical patterns of sports.

Luck is usually defined as the uncertainty in the outcome of games, that is, the randomness of game results [6,9,10,11]. However, luck has also been defined as the difference between the expected outcome and the actual outcome of a game [7,12]. At first glance, the two definitions seem completely different, yet they have largely the same meaning in practice. The expected outcome represents the power rating of the teams before a contest, which can be based on an official (e.g., the Elo rating system in chess) or an unofficial (e.g., bookmaker odds) rating system. In sports, when the probable winner wins against its “underdog” opponent, it is not exhibiting luck, because the results align with the expected results. The amount of luck involved in a sport or specific format of a game depends on how often, and by how much, an underdog team can prevail over their probable-winner opponent. Therefore, the difference between the expected and actual outcome is the randomness, that is, the uncertainty, in the outcome of a game, which for most spectators, is a very important part of watching games [4].

The fundamental purpose of our study was to measure and compare the two basketball forms in terms of luck. The idea for this study was motivated by the recognition that the two forms of basketball are very similar, but at the same time, very different. Our primary interest was exploring if the characteristics of 3 × 3 basketball (the reduced field size, the shorter available time for an attack, and the other differences compared to 5v5) increased or decreased the randomness in the outcome of games. Could the intensity of 3 × 3 basketball influence the luck involved? Is there a difference in terms of luck between the two forms of basketball?

Our first hypothesis is as follows:

**H1:**  *We expected there to be more luck involved in 3 × 3 basketball than in 5v5*.

Considering the literature, we assume this hypothesis for two main reasons. Firstly, the playing time in 3 × 3 is shorter and the game is more intense [13,14,15,16]; therefore, fewer periods are available for teams to develop rational game strategies. Without rational game strategies, the teams’ performances tend to fluctuate more, which could lower the skill part of the “luck–skill continuum” [7]. Secondly, 3 × 3 is an outdoor game, and teams do not adapt equally and consistently to adverse weather conditions. The outdoor factor could produce inconsistency in the results, which may increase the luck involved compared to the 5v5 form.

Furthermore, luck could differ not only for the two basketball forms but for the two sexes as well. Our second line of inquiry, then, was exploring whether there was a sex difference between or within each form of basketball in terms of luck. As previous studies have shown, there are sex differences in physical fitness [17], in the number of high-intensity sprint distances, and in the ratio of accelerations to decelerations [18], as well as in the ratio of blocks, steals, and missed two-point field goals [19]. These differences are all related to performance between the sexes, and thus may also affect the degree of luck. Moreover, as recent studies point out [20,21], the neglect of women’s sport in science is a serious problem, as we know essentially nothing about this aspect of sport. This is also the case for studies on luck, which have mostly been conducted in men’s sports, even though as the aforementioned studies show, there are significant sex differences in sports.

Only one recent study has investigated and compared the performance of male and female golfers and the role of luck [22]. The study highlights the lack of data on female golfers, which may partly explain the one-sided analysis of previous studies. However, basketball is an adequate sport from this point of view, as both sexes play the same kind of competitions, so the degree of luck can be analyzed well. Based on these considerations, our second research question is as follows: are sex-specific results equivalent in the two forms of basketball?

Our second hypothesis is as follows:

**H2:**  *We expected less luck to be involved in the women’s competitions compared to the men’s competitions*.

We also base our second hypothesis on two reasons. First, men’s competitions are usually more balanced; the power relations are not as straightforward for women [23,24]. Consequently, the more balanced the competition is, the more influence luck has on the results [7]. Second, women are about 3% more consistent in their free throw success than men are [25].

The comparison of team sports based on luck is not unique; several attempts have already been made on this subject [2,5,6,7]. However, our study has many novelties compared to the previous papers. On the one hand, luck in 3 × 3 basketball and the contrast between the 3 × 3 and 5v5 forms have never been analyzed. We created a new method to compare the two basketball forms in terms of luck. On the other hand, the depth of research is another novel contribution of our paper; we utilized four distinct methods to quantify luck by both form (3 × 3 and 5v5) and sex (male and female). In our study, we analyze the luck from the results of basketball World Cups, held between 2010 and 2019, in either format (3 × 3 and 5v5) and for both sexes. The aim of our study is not to examine all dimensions of basketball performance, but only to measure and compare the luck of the outcome, which can have many sources: form and sex.

Our study could provide benefits to experts in various roles. The results could improve the understanding of the nature of 3 × 3 basketball, which is gaining more and more interest thanks to the Olympic program. Firstly, 3 × 3 coaches could have a better grasp of their team and their players’ performances if they understood the differences in the effect of luck compared to that in 5v5 basketball. Secondly, players could see their ability more clearly and could evaluate their performance more realistically if they are aware of the influence of luck. Finally, decision-makers in sports at different levels could use our results to set realistic goals for reaching the world championships or the Olympics.

## 2. State of the Art of Explaining Winning Due to Luck

The number of papers published on the topic of 3 × 3 basketball is limited but has been growing in recent years. The main purpose of these studies is similar to ours: to understand the nature of 3 × 3 and how the game differs from the 5v5 form. We consulted the previous literature to fulfil our main goal of finding an appropriate methodology for showing whether there is a difference in uncertainty and luck between the sexes and between game types. We expected that the previous studies on 3 × 3 basketball could help us formulate our assumptions about the discrepancy in luck. Analyzing the research on uncertainty and luck should enable us to find the right methodology for our measurement.

Compared to the 5v5 form, there are significantly more ball contacts on average in 3 × 3 games, but no significant differences between the two forms were found in the average heart rate activity [16,26] or intensity (i.e., moderate to vigorous) of the activity [26]. Yet, the results of more recent research refute the latter statement. According to Herrán et al. [13] and Willberg et al. [16], the 3 × 3 form demands more movement, faster running speeds, and higher intensity for both acceleration and deceleration. Additionally, there are technical–tactical differences between the two formats [27]. A possible explanation of these findings is that the smaller number of participants per team in 3 × 3 tends to increase the relative space of a player [13]. Montgomery and Maloney [15] strengthened the argument for the higher physical demands of 3 × 3. They found that high-speed inertial movements within a limited area are required in 3 × 3 games, and as a result, a relatively high physiological response is created. The higher intensity and the narrower relative space of a player describes a high-pressure decision-making situation [28]. Players do not have enough time to choose the optimal decision [29,30]; therefore, a player’s inconsistent behavior adds variation to the performance of the team, which indicates there should be more luck involved in 3 × 3 basketball.

To understand the concept of luck, the following background is helpful. The notion of uncertainty originates from competitive balance studies that have focused on what has happened to competitive balance over time in sports leagues and what the consequences are for the business of sports [31,32,33]. The uncertainty of an outcome is a by-product of competitive balance, which is most prevalent as a hypothesis of outcome uncertainty in sports economic research [34,35]. According to the uncertainty-of-outcome hypothesis, the more uncertain the outcome of a contest, the greater the demand for it [36]. The luck research also uses the terminology of the uncertainty of outcome; however, some have aimed to compare the “pure” randomness in sports games without the sport demand context [7,9]. Further, Elias et al. [9] argued that uncertainty (or randomness) is a game feature “that [causes] the game to move from one state to another in an unpredictable (to the players) way” (p. 142). In sports, uncertainty is the sum of random elements in games, such as the weather or lucky bounces [9].

The underlying assumption of the measurement of luck is that every sport can be compared to another along with skill and luck [2,5,6,7]. A sport in which the expected players or teams always win is skill-based; luck, good or bad, does not tend to influence the outcomes of the competitions. However, the more skilled the players or the teams are, the more luck matters; this phenomenon is called the “paradox of skill” [6,7].

Because of the number of observations (a season contains multiple games) and the lack of changes in participants (same teams within the season), team sports are ripe for comparisons from the perspective of luck. Studies published on this topic have presented evidence that basketball is one of the team sports that is least affected by luck [2,5,6,7]. All these studies applied the notion of expected and actual outcomes to measuring luck. Some studies explained the low level of luck in basketball with the number of games in a basketball season, which is usually more comparable to other team sports [5,6]. Other studies offered the structure of matches as the main reason behind the low level of luck [2,5,7]. The scoring opportunities are the highest in this sport, which makes winning for a less skilled team without luck rather difficult. Mauboussin [7] highlighted that only 10% of the performance of basketball teams over a season can be explained by luck.

To compare different sports at different time points within or between seasons, the same objectives and unbiased indicators should be used [2,3,7]. Any ranking system requires a ranking methodology that is not without biases [7]. To this end, the expected outcomes are usually defined as the win fractions of teams in a season, which is easy to compare [2,5,6,7]. From this point, including luck into the rank estimates requires a calculation of the uncertainty of outcomes.

One of the simplest methods was presented by Mauboussin [7], who calculated the contribution of luck to the season in a sports league with the variable “ratio of luck” (variance of random win percentages) and the variable “variation of the observed winning percentages”. The other straightforward solution is to explain a contest’s results with the outcomes of the previous contest with control variables [3]. The lower the normalized mean square errors of the model, the more predictable the sport is, and, therefore, the less luck is involved in the sport. Getty et al. [5] contrasted the first-half win fractions with the second-half win fractions in a season, where the first and second halves were used as expected and actual outcomes. The higher the discrepancy within the season, the more luck influenced the results. Aoki et al. [2] quantified luck with a method similar to Mauboussin [7]. They created a coefficient that measures the distance between the observed final results of a sports league and an idealized perfectly balanced competition that demonstrates perfect skill. The coefficient is the indicator of luck, the difference between the perfect skill-based, and the observed outcomes. Finally, the last method belongs to Gilbert and Wells [6], who measured the fluctuation of a player’s performance from game to game, relative to the spread of the players’ skill levels.

Another approach was introduced by Groot and Groot [37]. They invented the Surprise Index, which is an alternative to the conventional indices for competitive balance in football. A team is given two “surprise points” if it beats its opponent as an underdog (lower ranked) and one point is awarded when the game ends in a tie. The Surprise Index is simply the ratio of the number of realized surprise points to the maximum number of surprise points. Like the rest of the luck-measuring methods, the Surprise Index is very data intensive because it needs game-by-game information [38]. The competitive balance is outside the scope of our paper; however, the Surprise Index can also be used as a measure of luck.

## 3. Methodology

It is essential to use perfectly balanced and identical types of data if the aim is to compare luck between sports [2,3,6,7]. The formats of World Cups in 3 × 3 and 5v5 basketball are analogous; they begin with the group stage and are followed by the knockout stage. Therefore, these competitions fully meet the precondition of comparison.

To measure luck, we applied four different methodologies. In line with theory, all the luck measurement methods were based on the expected and actual outcomes of the games. As in previous studies [2,7], the final rankings of the World Cups were used as the expected outcomes. The final rankings show the real power ranking of teams. Consequently, this is the most objective option for comparing sports without adding bias to the measurement. An artificial ranking (e.g., betting odds), thanks to the bias arising from the methodology used in compiling the ranking, would make the analysis incomparable and the results regarding luck incomprehensible. The observed results were applied as the actual outcomes.

When the final ranking for a team in a given World Cup was higher (i.e., it finished the competition in a better place) than that of the opponent, we expected that team to win the game. Conversely, a team with a worse final ranking was expected to lose the game. We applied two discrete choice variables, defined as follows:

(1) $${\mathrm{EO}}_{i}=\{\begin{array}{cc} 1 & \mathrm{if}\;\mathrm{team}\;i\;\mathrm{is}\;\mathrm{expected}\;\mathrm{to}\;\mathrm{win}\;\mathrm{the}\;\mathrm{game}\\ 0 & \mathrm{if}\;\mathrm{team}\;i\;\mathrm{is}\;\mathrm{expected}\;\mathrm{to}\;\mathrm{lose}\;\mathrm{the}\;\mathrm{game} \end{array}$$

(2) $${\mathrm{AO}}_{i}=\{\begin{array}{cc} 1 & \mathrm{if}\;\mathrm{team}\;i\;\mathrm{won}\;\mathrm{the}\;\mathrm{game}\\ 0 & \mathrm{if}\;\mathrm{team}\;i\;\mathrm{lost}\;\mathrm{the}\;\mathrm{game} \end{array}$$

where *EO_i_* is the expected outcome and *AO_i_* is the actual outcome of games for team *i*.

We implemented four different methods to quantify luck in 3 × 3 and 5v5 basketball World Cups. Firstly, we developed a new method that is similar to those in earlier works [2,5], but we tailored it to the type of our data. Secondly, we applied Groot and Groot’s [37] Surprise Index to basketball. Thirdly, we tried to explain the World Cup results with the outcomes of previous contests, for both form and sex. Lastly, to be able to examine the statistical significance between forms and sexes, we developed a new competitive balance indicator where the standard deviation of the index represents the luck.

It should be noted that, like all other studies on the topic of luck, we have deliberately used a measure of luck that filters out various biases. Winning a match basically requires the team’s preparation (physical, tactical, etc.), which should be unchanged throughout a tournament. An unexpected tactical move or an injury to a key player can of course affect the result of a match, which by its very character can be good or bad luck. It is not luck if this tactical knowledge and physical preparation can be demonstrated over several matches. However, in this case, none of our methodologies take this into account when calculating luck.

### 3.1. Performance Indicator

To compare 5v5 and 3 × 3 basketball in terms of luck, similar datasets were needed. Previous studies that aimed to measure luck in team sports dealt with seasonal data, which is not available in the tournament format of world championships. Consequently, we had to implement a new method to measure luck. We created a performance indicator that measures a team’s performance compared to expectations. The following formula was applied to analyze the expected and actual outcomes in basketball World Cups:

(3) $$PI_{i,j}=\frac{\sum_{i=1}^{k_{i}}AO_{i,j}+k_{i}}{\sum_{i=1}^{k_{i}}EO_{i,j}+k_{i}}$$

where *PI_i,j_* is the performance indicator of team *i* in basketball form *j*, *AO_i,j_* is the actual outcome, *EO_i,j_* the expected outcome of games for team *i* in basketball form *j*, and *k_i_* is the total number of games of team *I*, *k_i_* was essential to weight the proportions of the actual and expected outcomes.

A team with *PI* = 1 has shown the expected performance. A team with *PI* < 1 has underperformed and a team with *PI* > 1 has overperformed, compared to the expectations. After calculating the performance indicators, we were able to compute the standard deviations with the following formula:

(4) $$SD=\sqrt{\frac{\sum {\left(PI_{i,j}-\bar{PI_{i}}\right)}^{2}}{N}}$$

where *SD* is the standard deviation of the performance indicators and *N* is the number of teams that participated in the competition. The measurement indicator of luck is basically the standard deviation of the teams’ proportions of the actual and the expected outcomes. The larger the difference in the performance indicators (*PI*), from 1 in either direction in a World Cup, the greater the surprise, that is, the luck.

### 3.2. Surprise Index

Secondly, following Groot and Groot [37] and Goossens [38], we applied the Surprise Index to measure luck in a different way. However, this index was designed for football leagues, so we had to modify the formula for basketball World Cups.

We had to define a new dependent variable that captures the difference between the actual and expected outcomes. Thus, we calculated the absolute difference (*AD_i,j_*) between the two variables.

(5) $$AD_{i,j}=\left|AO_{i,j}-EO_{i,j}\right|$$

The *AD_i,j_* is 0 if there was no difference in the actual and the expected outcomes of the game and 1 if there was a difference, so the game ended with a surprise. With the new surprise variable, we were able to create a Surprise Index formula for basketball World Cups, which measures the ratio of games that ended with a surprise to all possible surprises.

(6) $$\mathrm{SI}=\frac{\sum_{i=1}^{k_{i}}AD_{i,j}}{\sum_{i=1}^{k_{i}}k_{i}}$$

The Surprise Index (*SI*) ratio varies between 0 and 1. The *SI* of a World Cup is 0 if all the games ended as expected. Conversely, a World Cup with a *SI* of 1 had only unexpected results, that is, surprises in all matches.

### 3.3. The Probit Models

The studies that measured luck in basketball leagues performed their analyses using seasonal data. However, there are substantially fewer games in World Cups, so these methods would be difficult to apply to our dataset. Therefore, we had to use other methods for quantifying luck in 3 × 3 and 5v5 basketball forms.

The structures of the competitions in the Olympic Games are more similar to the basketball World Cups than to the league system. Csurilla et al. [3], in their study, explained that the Olympic performance was based on the nations’ previous results at the Olympic Games. Zero-inflated beta regressions were applied by sport, and the normalized mean square errors of the models were the luck-based noise factor. Nevertheless, in accordance with other studies with binary variables, we used a probit model instead of ordinary least squares (OLS) [39]. The following probit model was examined:

(7) $$Y_{i,j}^{*}=\beta_{0}+\beta_{1}EO_{i,j}+\varepsilon_{i,j},\;\;Y_{i,j}^{}=1[Y_{i,j}^{*}>0]$$

where $Y_{i,j}^{*}$ is the actual outcome and $EO_{i,j}$ is the expected outcome of game *i* in basketball form *j* and $\varepsilon_{i,j}$ is the unexplained variance of the model in basketball form *j*. In line with the theory, the two basketball forms by sex were compared on the basis of the pseudo *R*^2^s, the goodness-of-fit measures of the models [40]. The higher the magnitude of the pseudo *R*^2^ is in a basketball form, the less luck is involved. The probit models were run separately for basketball forms and sexes.

### 3.4. Relative Score Difference Index

Ultimately, we intended to test whether there are statistical differences between the basketball forms and sexes because the methods described above are not suitable for that purpose. Therefore, we invented a new competitive balance measure whereby the standard deviation represents the amount of luck.

The Relative Score Difference Index (*RSDI*) is the ratio of point differences in a game to the difference in the final ranking of the teams. It can be calculated using the following formula:

(8) $$RSDI_{i,j}=\frac{\Delta TP_{i,j}}{\Delta FR_{i,j}}$$

where $\Delta TP_{i,j}$ is the total point difference and $\Delta FR_{i,j}$ is the final ranking difference of teams in game *i* and in basketball form *j*. The *RSDI* presents the point difference in a game relative to the position occupied in the final ranking. If a competition is balanced, close outcomes are expected in the games. Therefore, the lower the mean *RSDI* is in a tournament, the more the competition is balanced. After calculating the *RSDI*s, we were able to compute the standard deviations with the following formula:

(9) $$SD=\sqrt{\frac{\sum {\left(RSDI_{i,j}-\bar{RSDI_{i}}\right)}^{2}}{N}}$$

The standard deviation of the *RSDI* indicates the measure of luck in a competition. The explanation is straightforward: the more the *RSDI* varies in a sport, the more surprises arise in the outcomes of the games.

Finally, with the *RSDI*, we were able to test the differences between luck levels by form and sex. For this, we used a two-way analysis of variance (ANOVA).

## 4. Data

We collected the data from the game results of basketball World Cups between 2010 and 2019 from the websites of the International Basketball Federation (FIBA). The dataset contained altogether 12 tournaments, three World Cups in each form (3 × 3 and 5v5) and sex. The data from the World Cups held in 2010 were on the archive site of FIBA (archive.fiba.com); the rest can be found on the current web page (www.fiba.basketball). Table 1 presents the descriptive statistics of the data.

The final rankings of the teams (final ranking A, final ranking B variables) were applied to calculate the expected and actual outcomes (expected outcome, actual outcome variables) of the games. The *PI*, *SI*, and probit models were run using these variables. For the *RSDI*, the point differences of the games (using team A points, team B points variables) and the difference in the final rankings of the teams (using final ranking A, final ranking B variables) were applied.

## 5. Results

Before presenting the results of each method, we present the correlations between the different measures. The results of the correlation analysis (Table 2) indicate that all methods partly measure the luck in different ways. The results of the *PI* and the *SI* are almost identical (0.998), and these two measures have the highest correlation. The results of the probit models and the *RSDI* also correlate highly (−0.960). The largest difference proved to be between the results of the *PI* and the *RSDI*.

We carried out the analysis of the performance indicators and the Surprise Indexes first. Thereafter, we estimated the probit models, and finally, we tested the statistical differences with an ANOVA. The results of the methods are presented in the same order.

### 5.1. Performance Indicator and Surprise Index

As we expected, the men’s tournaments proved to be more influenced by luck than the women’s tournaments (H_2_). The standard deviation of the performance indicators was the highest for the 5v5 men’s basketball World Cup in 2014. Interestingly, the lowest value was zero. In the 3 × 3 women’s World Cup in 2019, every actual outcome was the same as our expectation based on the final outcomes. The Surprise Indexes showed analogous results but with different magnitudes (Table 3).

For our research question, we compared the performance indicators on the basis of the means of the standard deviations and the Surprise Indexes on the basis of the means in the basketball forms and sexes (Table 4). The means of the performance indicators’ standard deviations and Surprise Indexes were lower in the case of women in both basketball forms (0.047 and 0.049). The results imply that there were fewer surprises in the women’s World Cup competitions than in the men’s competitions, especially in the 3 × 3 basketball form.

### 5.2. The Probit Models

Similar to the results of the performance indicators and Surprise Indexes, the results of the probit models by the basketball form and sex suggest that luck had the most remarkable role in the men’s 3 × 3 World Cups, and the women’s competitions showed less luck compared to the men’s games; the pseudo *R*^2^ of the probit model was the lowest for this form (0.446). Conversely, the women’s 3 × 3 World Cups had the highest pseudo *R*^2^ (0.719). Luck played the smallest role in these tournaments, and the expected outcomes of the games explained the actual outcomes in the women’s 3 × 3 World Cups quite accurately. All probit models and coefficients were significant at the 1% level (Table 5).

### 5.3. Relative Score Difference Index

The results of the *RSDI* are slightly different from those of the previous methods (Table 6). According to the means of the *RSDI*s, the competition was the most balanced in the women’s 5v5 basketball World Cups (1.773), followed by the women’s 3 × 3 (2.002). The men’s results follow in the same order (4.275 and 6.816). The standard deviations of the *RSDI*s were completely identical in sequence to the means, which indicates that the most luck was involved in the 3 × 3 men’s basketball and the least in the women’s 5v5.

The two-way ANOVA also showed statistical differences between the basketball forms and sexes (Table 7). The two forms, 3 × 3 and 5v5, presented the highest statistical difference followed by sex, both at the 1% significant level. The discrepancies in variation were also statistically significant in the four different forms by gender, but only at the 5% level. In conclusion, we can argue that there is a disparity in basketball forms and sexes regarding luck, and the differences are statistically significant at the 5% level.

## 6. Discussion

The main purpose of our study was to determine if luck makes a difference in the match final results for different basketball forms (3 × 3 and 5v5) and sexes in World Cup games. Overall, four different measurement methods—partly based on the literature, partly on our own new ideas—were applied.

The results of the performance indicators, Surprise Indexes, and the goodness-of-fit of the probit models were completely identical in terms of the sequence: The 3 × 3 women’s World Cup had the lowest level of unexpected results based on the final ranking of teams. The women’s 3 × 3 was followed by the women’s and men’s 5v5 basketball form. The highest luck was associated with the 3 × 3 men’s World Cups.

The results of the *RSDI*, however, slightly differ from those of the other measures, and the significance of the difference could be tested in this case alone. The correlation analysis also showed the different results, and in this case, the relationship was only moderately strong. For the *RSDI*, another data point was applied (total points of a game) in addition to the ranking of the teams. This led to slightly different results compared to the previous ones. The standard deviation of the *RSDI*, which represents the amount of luck, was the lowest in women’s 5v5 basketball, followed by the women’s and men’s 3 × 3 in the same order. The results of the ANOVA test showed statistical differences in both the form type and sex, at least at the 5% significance level. Given the results of the test, we can suggest that nations that are chasing basketball success should focus on the women’s World Cups, especially in the 5v5. In this form, luck has the least effect on the results.

A correlation analysis has clearly confirmed that our methods for measuring luck are robust. *PI* and *SI* measure luck almost identically, thus in the future, the results will be comparable for either method. The probit method is a more sophisticated statistical method, as it uses regression to estimate luck. Its results are also highly correlated with the two indicators (*PI* and *SI*). Based on these results, we can claim that if we only consider the expected and the actual outcome of games, the methods used to measure luck in sports will give similar results. The slightly weaker but still strong correlation with the *RSDI* compared to the other indicators is due to the introduction of discarded points. The points scored in the games add another dimension to the luck formula, which is the difference in performance between the teams. In the future, a deeper, even match-level, examination of the *RSDI* may be worthwhile to see what accounts for the greater and unexpected point differences.

Our analyses confirmed all our hypotheses (H_1_ and H_2_). On the one hand, we expected higher luck to be involved in 3 × 3 than in 5v5 basketball, and the ANOVA test of the *RSDI* clearly demonstrated that assumption. The specific characteristics of 3 × 3 games, for example, their intensity [13,15,16] and their outdoor courts, tend to increase the unpredictability of this form. We could not distinguish which characteristic accounts for the difference, but this was not within the scope of our study. On the other hand, higher luck was expected in the men’s competitions and this hypothesis was also obviously confirmed. The men’s competitions were more balanced (see the results of the *RSDI*), and this tended to lead to higher luck in the competitions [7]. This finding is in line with previous studies that have found greater competitive balance in men’s contests [23,24].

There could be several possible explanations for our findings. Firstly, a player’s performance has much more weight in the results of the game in 3 × 3 compared to 5v5. Therefore, an outstandingly good or bad player in 3 × 3 can have a more decisive influence on the outcome of a tournament. Secondly, the 3 × 3 form is still too new, and nations have only just begun to realize its importance, as it just became an Olympic sport. The immense difference between men’s and women’s 3 × 3 basketball could be explained by the distinct approaches taken by men’s and women’s teams. A few nations (e.g., China, Hungary, France, and Russia) have started to focus on the 3 × 3 women’s World Cups in recent years and the power relations of teams are more unequivocal. On the men’s side, however, Serbia was the only nation that was able to perform at nearly the same level year after year. We assume that the difference between the two sexes will probably decrease over time, as in 5v5. After a few years, we will be able to analyze the stability of luck values in the 3 × 3 form.

Furthermore, in 5v5, the team is more important than any individual; this complexity tends to lead to more unexpected results. The difference in sexes is marginal; however, by each assessment method, the women’s 5v5 is expected to be more predictable. A possible explanation for this could be the paradox of skill phenomenon, which states that the more balanced the competition is, the more influence luck has on the results [7]. Men’s competitions are usually more balanced compared to women’s contests [23,24]; consequently, this difference in luck could be associated with the paradox of skill phenomenon. Those scientific comparisons are in line with personal observations including those from some of the authors’ own professional basketball experiences. There is a gap relating to the physical and technical knowledge in 3 × 3 games between women’s and men’s basketball teams. The differences between the team players are bigger in women’s teams, which is the case in women’s 5v5 as well. In the case of men, the differences between the team players are smaller.

## 7. Conclusions

The potential limitations of the findings must be mentioned. Measuring luck based on the final rankings of World Cups is novel, but it has drawbacks as well. Using the final rankings as the expected outcome does not consider the single-elimination part of the tournaments because the outcomes will always coincide with the actual results in the knockout phases. Nevertheless, the final rankings are the only way to compare different basketball forms by sex in terms of luck. Moreover, the number of games in a World Cup tends to have a major effect on the predictability of outcomes and, as a result, on luck. The men’s 5v5 basketball World Cups contain the highest number of games. The burden of a long tournament requires more strategy and tactics from a team to maintain the requisite performance during the whole competition. Performance optimization could lead to deliberate loss of matches, and the unexpected results tend to influence the level of luck.

Furthermore, we did not consider the effect of fluctuations in team strength on a tournament, which tend to act as luck. In the case of a basketball World Cup—which lasts for couple of weeks at most—the fluctuation in team strength is almost impossible to capture. Rather, we can talk about performance retention, which exists when the last group match—whose result does not matter for one of the teams—is used for rest. This phenomenon certainly gives some distortion to the calculations; however, it is difficult to prove when it has happened. The *RSDI* may be most affected by performance retention because it also uses the difference in points, which makes the *RSDI* more sensitive in the case of a very unexpected result. That could be a possible explanation for the different sequence in the results with the *RSDI*.

Finally, the contributions of our research should be addressed briefly as well. Only a limited number of studies have been conducted on 3 × 3 basketball; therefore, this analysis alone could further contribute toward the understanding of the nature of this version of basketball. The results may provide many benefits to experts in various fields, from researchers to professional basketball players. Firstly, luck is usually associated with predictability and the field of sports analytics, and data-based sport results prediction are seeing growing interest in the academic world [8]. Secondly, basketball coaches may better understand the differences between the two forms and sexes regarding luck if they are aware that the 3 × 3 game and men’s competitions are usually more influenced by luck. Thirdly, basketball players could make a more realistic self-performance evaluation if they were to understand that the results in 3 × 3 basketball games are less related to their performance compared to 5v5. Ultimately, our findings could be useful for decision-makers in sports governance as well. If a nation wants to go to the Olympics or world championships, it should target women’s competitions and within them, the 5v5 form of basketball. Because there is no difference in the number of medals that can be won, neither by form nor by sex, which would otherwise be an important aspect in the length of success [41]. If adequate support is allocated effectively for women’s teams, especially for the 5v5 women’s team, countries will be more likely to win a medal than if they supported the men’s teams.

In our study, we do not claim that winning in basketball is a matter of luck. It is a very small but important factor moderating success. Winning depends primarily on the tactical and physical preparation and the differences in the quality of both individual and team performances. Luck, however, matters when the teams are very similar in all other respects, as they are in the World Cup’s best-ranked teams. Here, luck can play a decisive role.

## Figures and Tables

**Table 1:** Descriptive statistics of the variables used.

Variable	*M*	*SD*	Min	Max
Year			2010	2019
Form (0 = 5v5, 1 = 3 × 3)	0.432	0.496	0	1
Sex (0 = men, 1 = women)	0.411	0.492	0	1
Team A points	51.503	31.977	4	129
Team B points	46.593	30.861	2	119
Final ranking A	8.668	6.539	1	32
Final ranking B	11.611	7.005	1	32
Expected outcome	0.679	0.467	0	1
Actual outcome	0.673	0.470	0	1

Note. *N* = 666 for all variables.

**Table 2:** Correlation matrix of the results of different methods.

	*PI*	*SI*	Probit	*RSDI*
*PI*	1			
*SI*	0.998	1		
Probit	−0.859	−0.885	1	
*RSDI*	0.685	0.724	−0.960	1

Note. *PI* = performance indicator; *SI* = Surprise Index; Probit = probit models; and *RSDI* = Relative Score Difference Index.

**Table 3:** The standard deviations of the performance indicators, the Surprise Indexes, and the total number of games for teams in 3 × 3 and 5v5 basketball World Cups between 2010 and 2019.

Year	Form	Sex	*SD*	*SI*	*N*
2010	5v5	Men	0.066	0.050	160
2010	5v5	Women	0.062	0.081	124
2014	5v5	Men	0.105	0.158	152
2014	5v5	Women	0.067	0.056	72
2017	3 × 3	Men	0.072	0.063	96
2017	3 × 3	Women	0.027	0.021	96
2018	5v5	Women	0.085	0.094	64
2018	3 × 3	Men	0.078	0.083	96
2018	3 × 3	Women	0.077	0.125	96
2019	5v5	Men	0.042	0.043	184
2019	3 × 3	Men	0.091	0.125	96
2019	3 × 3	Women	0.000	0.000	96

Note. *SI* = Surprise Index.

**Table 4:** The total standard deviations of the performance indicators, the Surprise Indexes, and the total number of games for teams by basketball form and sex.

Form	Sex	*SD*	*SI*	*N*
3 × 3	Women	0.047	0.049	288
5v5	Women	0.072	0.077	260
5v5	Men	0.074	0.081	496
3 × 3	Men	0.081	0.090	288

Note. *SI* = Surprise Index.

**Table 5:** The results of the luck-measuring probit models based on the unexplained variance, separated by basketball form and sex.

Variable	(1)	(2)	(3)	(4)
	Men’s 5v5	Men’s 3 × 3	Women’s 5v5	Women’s 3 × 3
*AO*				
*EO*	2.815 ***	2.590 ***	3.203 ***	3.329 ***
	(0.251)	(0.360)	(0.437)	(0.378)
Constant	−1.426 ***	−1.221 ***	−0.992 ***	−1.314 ***
	(0.209)	(0.319)	(0.201)	(0.239)
Observations	248	144	130	144
Pseudo R2	0.566	0.446	0.650	0.719

Note. Standard errors in parentheses. *** z < 0.01.

**Table 6:** The total standard deviations of the Relative Score Difference Indexes and the total number of games for teams by basketball form and sex.

Form	Sex	*M*	*SD*	*N*
5v5	Women	1.178	1.773	144
3 × 3	Women	1.531	2.002	144
5v5	Men	2.508	4.275	248
3 × 3	Men	4.303	6.816	130

**Table 7:** The results of the analysis of variance performed with the Relative Score Difference Indexes.

Source	*df*	*F*	*p*
Model	3	15.15	0.000
Form (3 × 3, 5v5)	1	37.48	0.000
Sex (women, men)	1	10.29	0.001
Form * Sex	1	4.63	0.032

## Data Availability

Publicly available datasets were analyzed in this study. This data can be found here: https://www.fiba.basketball/.
